# Laparoscopic Retrieval of a 13-Year-Old Retained Iatrogenic Metallic Foreign Body from the Pelvis: An Uncommon Case Report

**DOI:** 10.1055/s-0043-1764124

**Published:** 2023-03-02

**Authors:** Deepak Rajput, Amit Gupta, Sruthi Shasheendran, Rishit Mani, Amoli Tandon, Shyam Karuppusamy Krishnasamy, Rohik Anjum T. Siddeek, Krishna Sai Bhukya, Sanketh Edem

**Affiliations:** 1 Department of General Surgery, All India Institute of Medical Sciences Rishikesh, Dehradun, Uttarakhand, India; 2 Department of General Surgery, University College of Medical Sciences, Delhi, India; 3 Department of General Surgery, All India Institute of Medical Sciences Patna, Bihar, India

**Keywords:** hysterectomy, laparoscopic removal, retained surgical item, case report

## Abstract

Retained surgical foreign bodies are unanticipated events culminating from inadvertent operating room errors and may cause severe medical and legal problems between the patient and the doctor. Here, we report detecting a surgical instrument fragment 13 years after an open abdominal hysterectomy in a quadragenarian during her evaluation of a month-old complaint of lower abdominal and right thigh pain. A computed tomography scan of the abdomen demonstrated a radio-opaque linear foreign body traversing the right obturator foramen with extension into the pelvis cranially and the adductor compartment of the right thigh caudally. The metallic foreign body, identified as a fragmented handle of a uterine tenaculum forceps with a slender sharp-tip hook, could be removed laparoscopically from the pelvis after a diagnostic laparoscopy, preventing significant complications. The minimally invasive approach enabled a smooth recovery, and the patient could go home on the second postoperative day.

The Canadian Patient Safety Institute defines retained foreign body (RFB) as a patient safety incident wherein an object is inadvertently left in a body cavity or surgical wound following a procedure. 1 Commonly reported foreign bodies that are forgotten in the abdomen include mops, sponges, pieces of broken instruments or irrigating sets, rubber tubes, guide wires, sharp objects such as needles, and malleable retractors. 2 Usually brought to notice by media companies, the cases involving RFB are rarely published as they may cause legal issues and defame the institute or practitioner. It makes health care workers hesitate to report errors for fear of losing their jobs or fear of some other form of reprisal. The symptoms are usually nonspecific, and some patients remain asymptomatic and are never discovered or documented. This report presents a rare case of a retained metallic foreign body showing after 13 years following an abdominal hysterectomy.

## Case Report

A 42-year-old married female came to the general surgery outpatient department with pain in the right lower abdomen and anterior aspect of the right thigh over 1 month. She described the pain as continuous, sharp, and nonradiating that exacerbated with the movements of the right lower limb. She, otherwise, denied any history of trauma, any chronic illness, or alteration in bowel and bladder habits. On further questioning, it was revealed that she had undergone an open abdominal hysterectomy 13 years back for menorrhagia with an uneventful postoperative period. Clinical examination revealed a suprapubic transverse scar healed by primary intention and tenderness on deep palpation in the right iliac fossa with no palpable abdominal lump. There was no neurovascular deficit in the right lower limb, and spine examination was unremarkable. Digital rectal examination, per vaginal examination, and systemic examination were unremarkable.

**Fig. 1:**
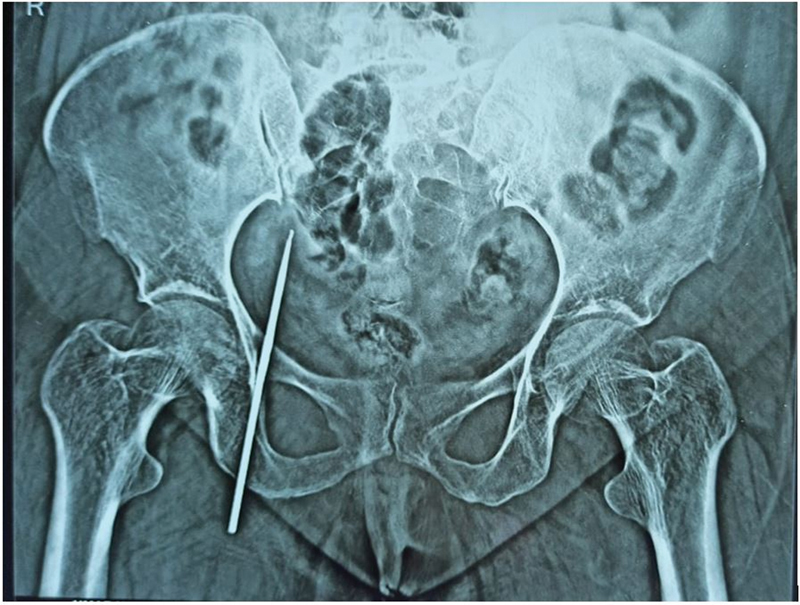
X-ray pelvis showing a linear radio-opaque shadow in the right hemipelvis.

**Fig. 2:**
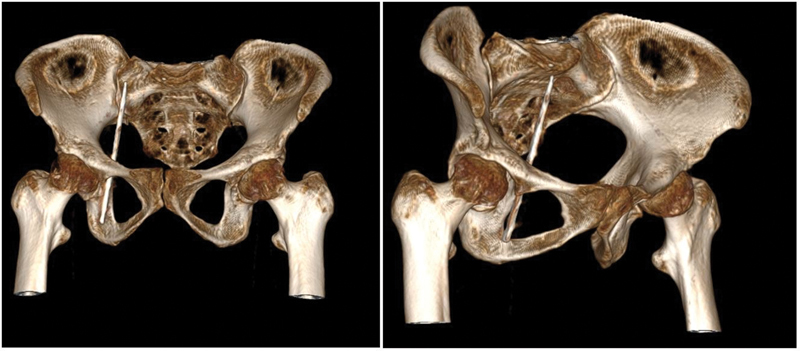
Reconstructed image from a tomography scan of the pelvis demonstrating an obliquely placed foreign body, with metallic attenuation, traversing through the right obturator foramen and tip at S1–2 vertebral level.

Laboratory values of complete hemogram, liver and renal function tests, urine, and blood sugar showed no deviation from the normal range. A pelvis roentgenogram revealed a linear radiopaque shadow with a tapering end lying obliquely over the right hemipelvis ( Fig. 1 ). Anticipating the foreign body as metallic, the patient underwent a contrast-enhanced computed tomography with angiography of the abdomen and pelvis, which demonstrated a metallic attenuation foreign body in the intermuscular plane of the adductor compartment of the right thigh, traversing the right obturator foramen with its tip lying at S1–2 vertebral level adjacent to the ileal loops ( Fig. 2 ). A three-dimensional reconstruction showed the upper half of the foreign body in proximity to external iliac vessels with no impingement.

**Fig. 3:**
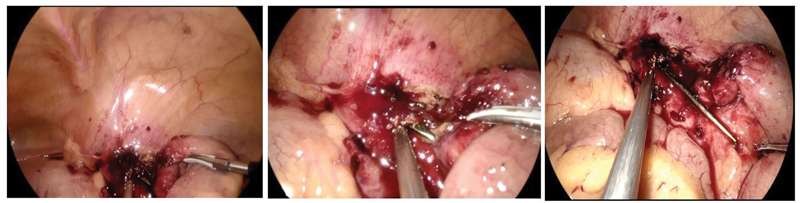
Laparoscopic view of the superior part of the foreign body that was hidden behind the adhered ileal loops.

**Fig. 4:**
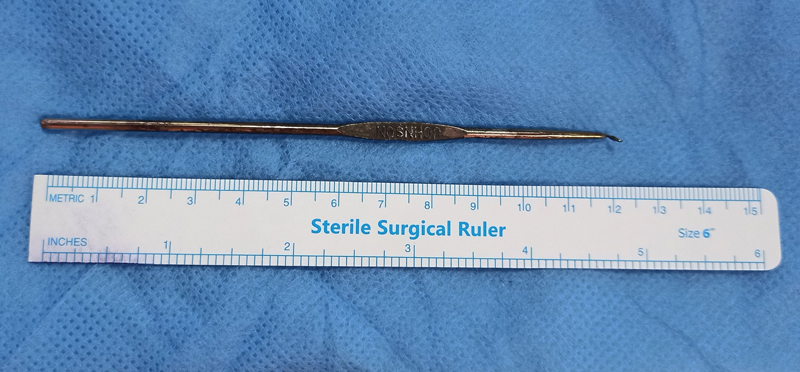
Retrieved linear metallic foreign body that had a slender sharp-tip hook, and was identified as one of the handles of uterine tenaculum forceps.

We proceeded with a diagnostic laparoscopy using a three-port position employed in transabdominal preperitoneal inguinal hernia repair. The ileal loops have adhered in the pelvis below the right medial umbilical ligament. A careful adhesiolysis revealed a metallic foreign body in the region of the right obturator foramen ( Fig. 3 ), which was extracted through the right-sided 10 mm port. The RFB was identified as a 5-inch long fragment of the uterine tenaculum hook used in gynecological surgeries to manipulate the cervix ( Fig. 4 ). The right thigh pain subsided on the same day following retrieval of the instrument. At the same time, the abdominal pain persisted for another 24 hours. The patient was sent home on the second postoperative day following an uneventful postoperative course. On long-term follow-up after 1.5 years, patient was doing her routine activities without any discomfort.

## Discussion

Retained sponges and instruments (RSIs) following surgical procedures present a unique problem for the surgeon. In most cases, the surgeon is held responsible for the errors of other members of the surgical team. This is the responsibility not only of the surgeon, but of the assistant(s) and operating theater nurses as well. 3 The nature of retained object varies from sponges to sharp and blunt instruments, needles, and threads. The clinical presentation ranges from an incidental finding on routine radiological evaluation to catastrophic complications depending on the type of foreign body reaction. The first type of bodily reaction is an aseptic fibrous response, which results in the formation of a granuloma, which can later be calcified and decomposed. The second is an inflammatory response that causes an abscess. 4

It has been estimated that one case of a retained item postsurgery occurs at least once a year in any hospital where 8,000 to 18,000 major procedures are performed annually. Studies evaluating RSI rates showed sponges accounted for the bulk of retained objects (69%) compared with instruments (31%). The abdomen, pelvis, vagina, and thorax accounted for common sites of RSI in decreasing order of incidence. 4 The time interval for detection varied from the operating room, the immediate postoperative period to several years following the procedure. In this case report the retained object remained undetected for a period of 13 years following the surgery. In the body's attempt to expel the RSI from the abdominal cavity, a myriad of presentations including bowel perforation, formation of fistulas, and intestinal obstruction (10–22%) can occur that may be fatal (0–2%). 5 6 Documents of primary surgery were unavailable and the surgeon could not be contacted. Patient was operated 13 years back when Surgical Safety Checklist published by the World Health Organization (WHO) had just been released and was coming into action in most centers but not all ( Supplementary Material S1 , online only). The possibility of this object being retained is that it was a part of another instrument and there is a possibility that the instrument broke during an unexpected torrential bleed and in an emergency setting went unnoticed by the surgeon. Patient had no postoperative complication hence the foreign body went unrecognized for 13 years.

Several etiological factors which could increase the operating room errors have been explored. These include open emergency surgery, long duration procedures, higher estimated blood loss, “after hours” surgery, change of surgical and nursing team during the procedure, and unanticipated or unplanned changes during the surgery. 7 8 Owing to the iatrogenic nature of the adverse event, several interventions to reduce this operating room error have been explored. The Surgical Safety Checklist published by the WHO in 2008 had provided the most promising results in preventing and reducing such errors. A 36% decrease in postoperative complications and mortality rates were observed on strict adherence to the checklist. 9 A meticulous manual mop and instrument count comprising single and dual count before and after the surgery respectively greatly reduced the chance of discrepancy in the counts. Novel methods combing technological advances with conventional counting systems like bar coding surgical sponges and radiofrequency detection system have showed promising results upon primary evaluation. 10

## Conclusion

Iatrogenic foreign bodies are avoidable adverse events following any procedure. A watchful eye for patients presenting with persistent or new symptoms postoperatively can aid in early detection. In asymptomatic RFBs cases, the patient should be informed and motivated for a reoperation. Strict adherence to the surgical safety checklist, meticulously performed and cross-verified manual mop counts, and adjuncts for verification of retained foreign objects can help reduce the incidence of RSI. A reduced RSI can help decrease patient morbidity and mortality, the excess financial burden on the health care system in terms of the additional expenses, litigations, and can reduce unforeseen complications.
