# Supplementary material for: Laparoscopic Retrieval of a 13-Year-Old Retained Iatrogenic Metallic Foreign Body from the Pelvis: An Uncommon Case Report
Source: Surg J (N Y). 2023 Mar 2;9(1):e62–6. doi: 10.1055/s-0043-1764124 (PMC9981328; doi:10.1055/s-0043-1764124)
Supplement: Supplementary file 1 — Supplementary Material [file 10-1055-s-0043-1764124-s2100162.pdf]

Supplementary Material S1

CARE case report guidelines CARE Checklist of information to include when writing a case report (cc) BY-NC-ND ✓

Topic	Item	Checklist item description	Reported on Line
**Title**	1	The diagnosis or intervention of primary focus followed by the words "case report"	page no.1
**Key Words**	2	2 to 5 key words that identify diagnoses or interventions in this case report, including "case report"	page no.5
Abstract	3a	Introduction: What is unique about this case and what does it add to the scientific literature?	page no.5
**(no references)**	3b	Main symptoms and/or important clinical findings	page no.6
	3c	The main diagnoses, therapeutic interventions, and outcomes	page no.7
	3d	Conclusion—What is the main "take-away" lesson(s) from this case?	page no.9
**Introduction**	4	One or two paragraphs summarizing why this case is unique (**may include references**)	
**Patient Information**	5a	De-identified patient specific information	page no.6
	5b	Primary concerns and symptoms of the patient	page no.6
	5c	Medical, family, and psycho-social history including relevant genetic information	page no.6
	5d	Relevant past interventions with outcomes	page no.6
**Clinical Findings**	6	Describe significant physical examination (PE) and important clinical findings	page no.6
**Timeline**	7	Historical and current information from this episode of care organized as a timeline	
**Diagnostic Assessment**	8a	Diagnostic testing (such as PE, laboratory testing, imaging, surveys).	Page no.6
	8b	Diagnostic challenges (such as access to testing, financial, or cultural)	
	8c	Diagnosis (including other diagnoses considered)	Page no.6
	8d	Prognosis (such as staging in oncology) where applicable	
**Therapeutic Intervention**	9a	Types of therapeutic intervention (such as pharmacologic, surgical, preventive, self-care)	page no.7
	9b	Administration of therapeutic intervention (such as dosage, strength, duration)	
	9c	Changes in therapeutic intervention (with rationale)	
**Follow-up and Outcomes**	10a	Clinician and patient-assessed outcomes (if available)	page no.7
	10b	Important follow-up diagnostic and other test results	page no.7
	10c	Intervention adherence and tolerability (How was this assessed?)	
	10d	Adverse and unanticipated events	
**Discussion**	11a	A scientific discussion of the strengths AND limitations associated with this case report	
	11b	Discussion of the relevant medical literature **with references**	page no.7,8
	11c	The scientific rationale for any conclusions (including assessment of possible causes)	page no.7,8

Supplementary Material S1 (*Continued*)

Topic	Item	Checklist item description	Reported on Line
	11d	The primary “take-away” lessons of this case report (without references) in a one paragraph conclusion	page no.9
**Patient Perspective**	12	The patient should share their perspective in one to two paragraphs on the treatment(s) they received	
**Informed Consent**	13	Did the patient give informed consent? Please provide if requested	**Yes** ☑ **No** ☐

 © 2023. The Author(s).
